# Decontamination of N95 masks for re-use employing 7 widely available sterilization methods

**DOI:** 10.1371/journal.pone.0243965

**Published:** 2020-12-16

**Authors:** Anand Kumar, Samantha B. Kasloff, Anders Leung, Todd Cutts, James E. Strong, Kevin Hills, Frank X. Gu, Paul Chen, Gloria Vazquez-Grande, Barret Rush, Sylvain Lother, Kimberly Malo, Ryan Zarychanski, Jay Krishnan

**Affiliations:** 1 Sections of Critical Care Medicine and Infectious Diseases, Departments of Medicine, Medical Microbiology and Pharmacology, University of Manitoba, Winnipeg, Canada; 2 National Microbiology Laboratory, Public Health Agency of Canada, Winnipeg, Canada; 3 National Centre for Foreign Animal Diseases, Canadian Food Inspection Agency, Winnipeg, Canada; 4 University of Toronto, Toronto, Canada; 5 Section of Critical Care Medicine, Department of Medicine, University of Manitoba, Winnipeg, Canada; 6 Occupational & Environmental Safety and Health, Winnipeg Regional Health Authority, Winnipeg, Canada; 7 Sections of Critical Care and Hematology, Departments of Medicine and Community Health Sciences, University of Manitoba, Winnipeg, Canada; VIT University, INDIA

## Abstract

The response to the COVID-19 epidemic is generating severe shortages of personal protective equipment around the world. In particular, the supply of N95 respirator masks has become severely depleted, with supplies having to be rationed and health care workers having to use masks for prolonged periods in many countries. We sought to test the ability of 7 different decontamination methods: autoclave treatment, ethylene oxide gassing (ETO), low temperature hydrogen peroxide gas plasma (LT-HPGP) treatment, vaporous hydrogen peroxide (VHP) exposure, peracetic acid dry fogging (PAF), ultraviolet C irradiation (UVCI) and moist heat (MH) treatment to decontaminate a variety of different N95 masks following experimental contamination with SARS-CoV-2 or vesicular stomatitis virus as a surrogate. In addition, we sought to determine whether masks would tolerate repeated cycles of decontamination while maintaining structural and functional integrity. All methods except for UVCI were effective in total elimination of viable virus from treated masks. We found that all respirator masks tolerated at least one cycle of all treatment modalities without structural or functional deterioration as assessed by fit testing; filtration efficiency testing results were mostly similar except that a single cycle of LT-HPGP was associated with failures in 3 of 6 masks assessed. VHP, PAF, UVCI, and MH were associated with preserved mask integrity to a minimum of 10 cycles by both fit and filtration testing. A similar result was shown with ethylene oxide gassing to the maximum 3 cycles tested. Pleated, layered non-woven fabric N95 masks retained integrity in fit testing for at least 10 cycles of autoclaving but the molded N95 masks failed after 1 cycle; filtration testing however was intact to 5 cycles for all masks. The successful application of autoclaving for layered, pleated masks may be of particular use to institutions globally due to the virtually universal accessibility of autoclaves in health care settings. Given the ability to modify widely available heating cabinets on hospital wards in well-resourced settings, the application of moist heat may allow local processing of N95 masks.

## Introduction

The COVID-19 pandemic is proving to be an exceptional stress on hospital and health systems resources around the world. Many countries are experiencing or imminently expecting shortages for a variety of equipment and disposable supplies. A tightening supply of N95 masks that allow for protection from airborne pathogens and aerosolized viruses including SARS-CoV-2 is of particular and immediate concern. Without an adequate supply of N95 masks, health care providers are at substantial risk of contracting COVID-19 during the course of their duties. The occurrence of patient to health care worker (HCW) spread of SARS-CoV-2 at sufficiently high rates would lead to demoralization of the workforce, depletion of HCWs for quarantine and would turn hospitals into hotspots for infection transmission.

N95 masks are normally single use products. However, according to news reports, extended use and re-use of N95 masks has occurred or is ongoing in multiple institutions in the United States, Canada, Italy and many other countries [1]. Persistent shortages may increase the re-use of N95 masks globally as the pandemic progresses.

We sought to determine whether a range of different N95 masks would retain structural and functional integrity after treatment with widely available decontamination techniques. Concurrently, we also determined the ability of each decontamination technique to effectively inactivate virus on experimentally inoculated masks.

## Materials & methods

Several different N95 respirator masks were assessed using standard autoclaving, vaporous hydrogen peroxide (VHP) exposure, peracetic acid dry fogging (PAF), ethylene oxide (EtO) gassing, low temperature hydrogen peroxide gas plasma (LT-HPGP) treatment, ultraviolet-C (UV-C) irradiation and moist heat treatment.

Four mask models, including VFlex 1804, Aura 1870, 1860 (3M Company, St. Paul, Minnesota) and AO Safety 1054S (Pleats Plus) Respirator (Aearo Company, Indianapolis) were subjected to all decontamination technologies for the purpose of performance testing as well as quantifying viral inactivation. Two additional respirator models, 3M 8210 and 9210 respirator models (3M Company, St. Paul, Minnesota) were included only for performance testing following decontamination. No valved-type masks were assessed.

EtO gas treatment was done using the model 5XLP Steri-Vac Sterilizer/Aerator (3M Company, St. Paul, Minnesota) with 1 hr exposure and 12 hr aeration time.

LT-HPGP treatment was performed using a STERRAD^®^ 100NX sterilizer (Advanced Sterilization Products, Irvine, California). This device generates hydrogen peroxide vapor from 59% liquid H_2_O_2_, which is then electromagnetically excited to a low-temperature plasma state. Highly reactive species are generated from the hydrogen peroxide vapor in this state to facilitate faster decontamination of medical equipment. A standard 47 minute cycle with 30 minutes of exposure time to the reactive species was used for the mask treatment. No aeration is required as part of the standard cycle.

VHP treatment was performed with the VHP^®^ ARD System (Steris, Mentor, OH), it uses 35% liquid H_2_O_2_ to generate hydrogen peroxide vapor. Two program cycles were used: A one hour cycle, consisting of 10 min dehumidification, 3 min conditioning (5 g/min), 30 min decontamination (2.2 g/min) and 20 min aeration; or a 5 hour cycle, consisting of 10 min dehumidification, 3 min conditioning (5 g/min), 2 hr decontamination (2.2 g/min), 2 hr dwell and 45 min aeration. Both Program cycles had peak VHP concentrations of 750 ppm.

For PAF, a dry fogging system using fogger head and nozzles purchased from Ikeuchi USA (Blue Ash, OH) was used as described elsewhere [2]. One tenth diluted Minncare Cold Sterilant, a liquid peracetic acid (Mar Cor Purification, Skippack, PA) was used. The fogger was run until the relative humidity rose to 80–90%, which required 30 ml of the diluted chemical. The fogger was then turned off and the masks exposed for 1 hr.

VHP and PAF treatments were conducted in a 40 ft^3^ glovebox (Plas Labs Inc. Lansing, MI).

Ultraviolet-C irradiation (UVCI) at a wavelength of 254 nm was delivered using an Asept.2X UV-C disinfection unit (Sanuvox Inc., St. Laurent, QC) at a distance of 86 inches/218 cm according to protocol described by Lowe et al [3] modified for local conditions. Assay of UV-C dose, measuring 400 mJ/cm^2^, was performed using a PM100A dosimeter (Thorlabs Co., Newton, NJ) and a S120VC light sensor (Thorlabs Co., Newton, NJ). Assessment of UV exposure was measured on a representative 3M 1870+ Aura above the outer layer, below the first layer, and beneath the thick middle layer of fabric using Photochromatic Ultraviolet-C (UV-C) Dosimeter Disks (Intellego Technologies, Stockholm Sweden).

Standard autoclaving was performed using an Amsco Lab 250 model (Steris Life Sciences, Mentor, OH) with a peak temperature of 121°C for 15 min; total cycle time was 40 min (10 min conditioning/air removal, 15 min exposure, 15 min drying/exhaust).

Moist heat treatment (MHT) was applied through the use of an OR-7854 warming cabinet (Imperial Surgical/SurgMed Group, Dorval, QC) set at 70°C and 75°C. Humidity was passively increased to 22% by placement of an open 2 gallon stainless steel container filled with hot tap water and a wet cotton towel draped to the base of the container to increase evaporative surface area. Temperature and humidity in the cabinet were confirmed with a model OM-EL-USB-2-Plus logger (Omega Environmental, St-Eustache QC).

### Effectiveness of decontamination

The ability of each decontamination technology to inactivate infectious virus was assessed using experimentally inoculated masks. Small swatches cut from one of each of the 4 respirator models was surface contaminated on the exterior with vesicular stomatitis virus, Indiana serotype (VSV) or SARS-CoV-2 (contaminated group). SARS-CoV-2 was only utilized if the decontamination method was available within the CL3 suite at Canada’s National Microbiology Laboratory. VSV was used if the decontamination method was only available outside the CL3 suite. The inoculum was prepared by mixing the virus in a tripartite soil load (bovine serum albumin, tryptone, and mucin) as per ASTM standard to mimic body fluids [4]. Ten μl of the resulting viral suspension containing an estimated 6.75 log TCID_50_ of VSV or 5.0 log TCID_50_ of SARS-CoV-2 was spotted onto the outer surface of each respirator at 3 different positions. Following 1–2 hr of drying, swatches from masks underwent each of the decontamination procedures. Corresponding positive control masks were concurrently spotted with the same viral inoculum, dried under the biosafety cabinet, and processed for virus titer determination to account for the effect of drying on virus recovery.

Following decontamination, virus was eluted from the mask material by excising the spotted areas on each mask swatch and transferring each into 1 ml of virus culture medium (DMEM with 2% fetal bovine serum and 1% penicillin-streptomycin). After 10 minutes of soaking and repeated washing of the excised material, the elution media was serially diluted in virus culture medium for evaluation in a fifty-percent tissue culture infective dose (TCID_50_) assay. 100 μl of each dilution was transferred into triplicate wells of Vero E6 cells (ATCC CRL-1586) seeded 96 well plate. At 48 hours (VSV) or 96 hours (SARS-CoV-2) post-infection, cells were examined for determination of viral titres via observation of cytopathic effect. Titres were expressed as TCID_50_/ml as per the method of Reed and Muench [5]. Results for each treatment indicate mean ± standard deviations of three biological replicates.

### Impact of decontamination on structural and functional integrity

A group of the N95 masks without viral contamination (clean group) underwent multiple decontamination treatments by all the decontamination methods.

Afterwards, these respirator masks were visually and tactilely assessed for structural integrity and underwent quantitative fit testing using a TSI PortaCount 8038+ (Shoreview MN, USA) to assess functional integrity. Fit testing was carried out on volunteer staff members who previously successfully fit tested for a given mask model. Masks were considered to be functionally intact if quantitative fit testing resulted in a fit factor of more than 100 for normal and deep breathing exercises [6,7]. For autoclaving, VHP, PAF and UVCI, we assessed integrity after 1, 3, 5 and 10 cycles; for LT-HPGP treatment after 1, 2, 5 and 10 cycles; for EtO gas treatment after 1 and 3 cycles; and for MHT after 3 and 10 cycles.

The filtration efficiency evaluation was conducted by SGS Lab (Grass Lake, Michigan, USA), following the ASTM testing conditions for particulate filtration (ASTM F2299 and ASTM F2100). Briefly, masks were individually packaged in labeled paper bags and overnight couriered to the testing facility. At the facility, aqueous suspensions of monodisperse Latex polystyrene beads at 0.1 μm were prepared for the challenge particles. Filtered and dried air was passed through a nebulizer to produce an aerosol containing the suspended Latex beads. The fit test sampling probes (TSI Incorporated, Shoreview, MN, USA) leftover from fit testing were sealed with hot glue. N95 filtering facepiece respirators (FFRs) were attached to a filter holder and placed between inflow and outflow tubes. The aerosol was passed through a charge neutralizer and mixed and diluted with additional preconditioned air to produce the challenge aerosol to be used in the test. The aerosol was fed (1.0 scfm) through the FFRs, and filtration efficiency was obtained using two-particle counters (Lasair^®^ III 110 Airborne Particle Counter, Particle Measuring Systems^®^, a Spectris company Boulder, CO, USA) connected to the feed stream and filtrate. Pressure differential (DHII-007, Dwyer Instruments International, Michigan City, IN, USA), airflow (M-50SLPM-D/5M, Alicat Scientific, Tucson, AZ, USA), temperature, humidity (HMT330 Humidity and Temperature Meter, Vaisala, Helsinki, Finland) and barometric pressure (PTU200 Transmitter, Vaisala, Helsinki, Finland) were also characterized in the experimental apparatus. Filtration efficiency based on the ASTM methodology was calculated as the persistent fraction of aerosolized 0.1 μm latex microbeads in air before and after passage through the N95 mask [8]. An N95 mask should filter a minimum of 95% of aerosolized particles of that size.

## Results

### Effectiveness of decontamination

Apart from UVCI, all the decontamination treatments assessed successfully inactivated the challenge VSV from all of the four mask materials in comparison to the untreated drying controls (Table 1). A demonstrable reduction of greater than six logs of infectious VSV was recorded for those respirator masks.

**Table 1:** Sterilization efficacy of decontamination methods.

Mean virus recovery post-decontamination (LogTCID_50_ ± SD) compared to drying controls									
Virus	Mask	Control	Autoclave	VHP*	PAF	EtO	LT-HPGP	UV-C	Moist Heat†
VSV	3M 1860	6.1 ± 0.3	0	0	0	0	0	2.2 ± 2.2	0
	3M Aura 1870	6.5 ± 0.8	0	0	0	0	0	1.8 ± 2.0	0
	3M Vflex 1804	6.4 ± 0.2	0	0	0	0	0	1.4 ± 1.5	0
	AO Safety 1054	6.5 ± 0.3	0	0	0	0	0	1.5 ± 1.4	0
SARS-CoV-2	3M 1860	4.4 ± 0.2	0	0	0	ND	ND	ND	0
	3M Aura 1870	4.5 ± 0.4	0	0	0	ND	ND	ND	0
	3M Vflex 1804	4.4 ± 0.4	0	0	0	ND	ND	ND	0
	AO Safety 1054	4.4 ± 0.4	0	0	0	ND	ND	ND	0

ND = not done, 0 = no growth.

VHP*: Vaporized hydrogen peroxide -VHP^®^ ARD System, 1 hour program cycle (VSV) or 5 hour program cycle (SARS-CoV-2).

Moist Heat†: 70°C with 22% relative humidity X 1 hr (VSV) or 75°C with 22% relative humidity X 3 hrs (SARS-CoV-2).

PAF: Peracetic acid dry fogging system, VHP = vaporous hydrogen peroxide, EtO = ethylene oxide.

LT-HPGP: Low temperature hydrogen peroxide gas plasma.

UV-C: Ultraviolet light-C radiation (254 nm wavelength).

TCID_50_: Median tissue culture infectious dose (per mL).

Control: Virus-inoculated mask materials subjected to air-drying only for 1–2 hrs.

**Fig 1:**
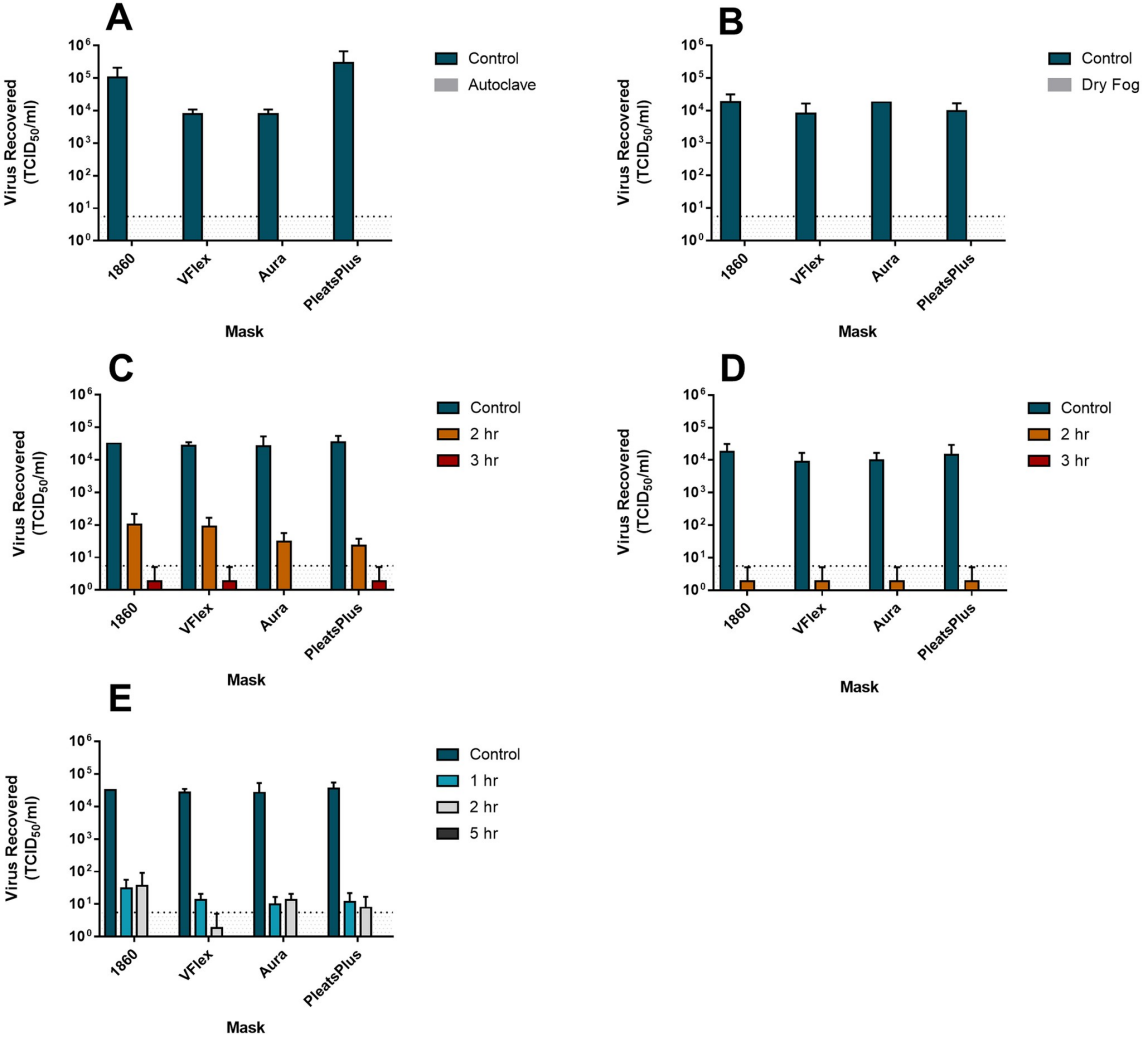
Inactivation efficacy of various decontamination methods against SARS-CoV-2 on experimentally contaminated N-95 masks. Coupons of inoculated N-95 masks (n = 4 models) were subjected to (A) autoclaving at 121°C for 15 minutes; (B) peracetic acid dry fogging; (C) exposure to 70°C + 22% RH for 2–3 hours; (D) exposure to 75°C +22% RH for 2–3 hours; and (E) VHP treatment for 1,2 or 5 hour cycle times. Virus recovery post-treatment was determined by elution of coupons in culture medium and endpoint titration in Vero E6 cells via TCID_50_ assay. Inoculated, untreated drying controls of each mask material were included as positive controls in all experiments. Results indicate means +/- standard deviations of three biological replicates. Dotted lines indicate quantification limits of the TCID_50_ assay.

Mask materials inoculated with SARS-CoV-2 had no recoverable virus following autoclaving and peracetic acid dry fogging treatments (Fig 1). While VHP decontamination led to complete inactivation of SARS-CoV-2, an extended cycle time was required compared to that of VSV (Table 1). Complete moist heat inactivation of SARS-CoV-2 was achieved with 3 hrs exposure at 75°C and 22% relative humidity (RH) (Table 1); any exposure of SARS-CoV-2 of less than 3 hrs at 75°C or with an exposure of 3 hrs at 70°C (both at 22% RH) resulted in a reduction of viral titre with residual recoverable virus (Fig 1). The titer of the starting SARS-CoV-2 virus was slightly lower than that of VSV, therefore the maximum demonstrated reduction was 4.5 logs. We could not validate the effectiveness of EtO and LT-HPGP against SARS-CoV-2 as they were not available at the National Microbiology Laboratory.

**Fig 2:**
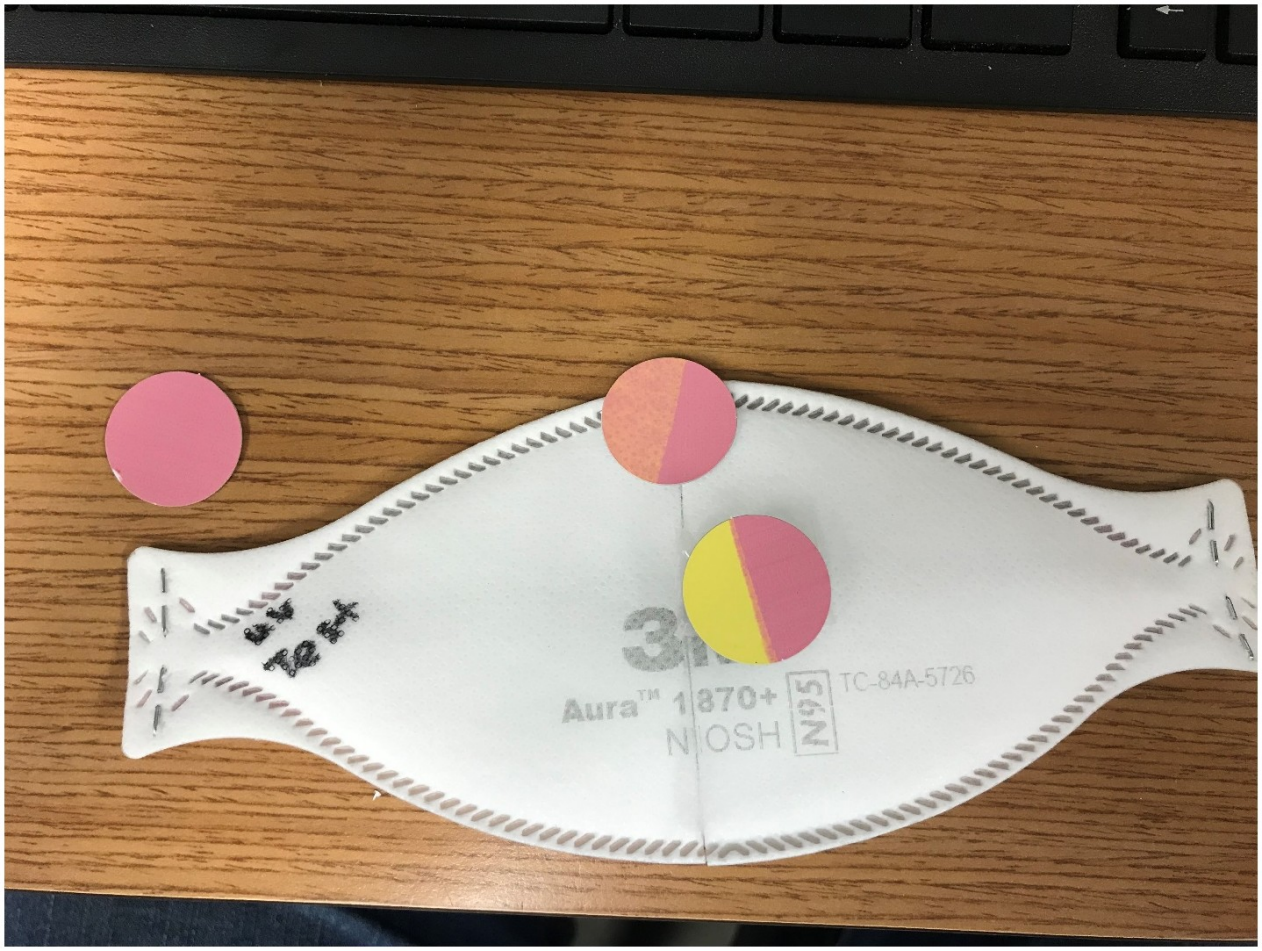
Penetration of UV-C through N95 masks. The degree of UV-C penetration through a layered N95 mask was demonstrated using photochromic UV-C Dosimeter Disks. A 3M 1870+ Aura respirator mask was cut in half, and dosimeter disks were placed directly on top (left disk), half-way under the first fabric layer (top center disk), or half-way under the thick middle layer (lower central disk) of material. A color change from yellow (unexposed) to deep pink was achieved on exposed portions of all disks. The lighter orange color, consistent with reduced UV-C exposure, was revealed on the disk partially covered by the top layer of mask material during UV-C treatment, while the disk placed half beneath the thick middle fabric layer showed no color change from yellow, indicating a lack of exposure to significant UV-C radiation.

Although several UVCI doses were assessed, only the highest dose is reported. For UVCI, a substantial and consistent decrease in virus titer (between 4 and 5 log) was shown; however, persistent viable VSV was isolated from each mask. The maximum delivered dose on each side of the masks was 560 mJ/cm2. A total dose of 1120 mJ/cm^2^ was delivered to each mask taking into account lamps placed on each side. Lower delivered doses similarly consistently showed persistent viable VSV of UVCI. A supplemental examination using disposable adhesive photochromatic UV-C dosimeter disks (“dots”) that exhibit a defined color changes with specific UV-C dose exposure demonstrated a failure of UV-C to penetrate through the middle of 3 layers of the 3M 1870 and AO Pleats Plus masks (see Fig 2).

In summary, all decontamination methods (except UVCI) resulted in no growth of virus in decontaminated specimens.

### Impact of decontamination on structural and functional integrity

All decontamination methods resulted in no significant change on visual or tactile inspection. In addition, all masks exhibited preserved structural and functional integrity of masks as assessed by fit testing for at least one cycle of treatment (Table 2).

**Table 2:** Quantitative fit testing results of N95 masks after repeat decontamination cycles.

PortaCount Result (normal & deep breathing exercises only)					
Groups	Masks				
Control	3M 1860	pass			
	3M Aura 1870	pass			
	3M Vflex 1804S	pass			
	AO Safety 1054S	pass			
	3M 8210	pass			
	3M 9210	pass			
		# of cycles			
		1	3	5	10
Autoclave	3M 1860	pass	fail	fail	fail
	3M Aura 1870	pass	pass	pass	pass
	3M Vflex 1804S	pass	pass	pass	pass
	AO Safety 1054S	pass	pass	pass	pass
	3M 8210	pass	fail	fail	fail
	3M 9210	pass	pass	pass	pass
		1	3		
EtO	3M 1860	pass	pass		
	3M Aura 1870	pass	pass		
	3M Vflex 1804S	pass	pass		
	AO Safety 1054S	pass	pass		
		1	2	5	10
LT-HPGP	3M 1860	pass	fail	fail	fail
	3M Aura 1870	pass	fail	fail	fail
	3M Vflex 1804S	pass	fail	fail	fail
	AO Safety 1054S	pass	pass	fail	fail
	3M 8210	pass	fail	fail	fail
	3M 9210	pass	fail	fail	fail
		1	3	5	10
VHP	3M 1860	pass	pass	pass	pass
	3M Aura 1870	pass	pass	pass	pass
	3M Vflex 1804S	pass	pass	pass	pass
	AO Safety 1054S	pass	pass	pass	pass
		1	3	5	10
PAF	3M 1860	pass	pass	pass	pass
	3M Aura 1870	pass	pass	pass	pass
	3M Vflex 1804S	pass	pass	pass	pass
	AO Safety 1054S	pass	pass	pass	pass
		1	3	5	10
UV-C (1120 mJ/cm^2^)	3M 1860	pass	pass	pass	ND
	3M Aura 1870	pass	pass	pass	ND
	3M Vflex 1804S	pass	pass	pass	ND
	AO Safety 1054S	pass	pass	pass	ND
	3M 8210	pass	pass	pass	ND
	3M 9210	pass	pass	pass	ND
		1	3	5	10
Moist Heat (75°C & 22% RH X 3 hr)	3M 1860	ND	pass	ND	pass
	3M Aura 1870	ND	pass	ND	pass
	3M Vflex 1804S	ND	pass	ND	pass
	AO Safety 1054S	ND	pass	ND	pass
	3M 8210	ND	pass	ND	pass
	3M 9210	ND	pass	ND	pass

PAF: Peracetic acid dry fogging system, VHP = vaporous hydrogen peroxide, EtO = ethylene oxide.

LT-HPGP: Low temperature hydrogen peroxide gas plasma.

UV-C: Ultraviolet light-C radiation (254 nm wavelength).

**Fig 3:**
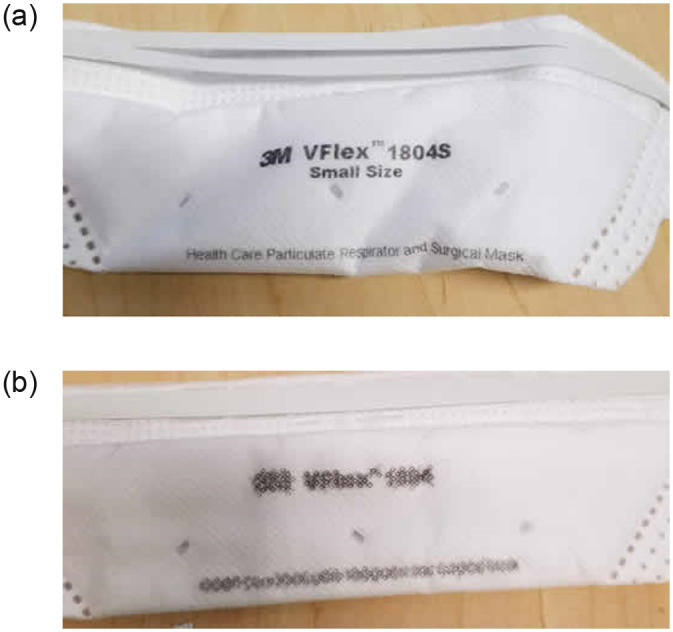
Impact of autoclaving on ink print on 3M 1804 filtering facepiece respirator. A single cycle of autoclaving consistently resulted in varying levels of ink bleeding on 3M 1804 and 9210 masks (a before; b after). Minimal further bleeding of ink was inconsistently observed after additional autoclave cycles. a) Before autoclave. b) After single cycle of autoclave.

The 3M 1870 Aura model exhibited some stiffness of straps with more than 16 cumulative hours of MHT. The 3M Vflex 1804 and 9210 as well as the AO Safety 1054 models exhibited some mild bleeding of the ink label upon autoclaving (Fig 3).

Autoclaving resulted in functional failure of the 3M 1860 and 8210 (molded) models after the first cycle but the other masks (all pleated, layered fabric models), retained integrity through 10 cycles, the highest number tested. All masks treated with EtO and UVCI retained integrity though 3 and 5 cycles respectively (maximum number of cycles tested). LT-HPGT-treated masks failed fit testing beyond the first cycle (5 of 6 respirators at after 2 cycles; 6 of 6 failures with 5 and 10 cycles) while VHP exposure, PAF and MHT maintained mask integrity through the maximum 10 cycles tested.

With a few exceptions, filtration testing demonstrated congruent deficiencies in filtration efficiency (Table 3). A filtration efficiency of ≥ 95% is considered consistent with the N95 designation. One exception was that the fit test failing masks in the autoclave group (molded mask models 3M 1860 and 8210) passed filtration efficiency testing. In addition, while all masks passed fit testing after a single cycle of LT-HPGP, half failed filtration testing at the same point.

**Table 3:** Filtration efficiency testing results of N95 masks after repeat decontamination cycles.

Filtration Efficiency (0.1 μm latex beads)					
Groups	Masks	% efficiency			
Control	3M 1860	99.8			
	3M Aura 1870	>99.9			
	3M Vflex 1804S	99.8			
	AO Safety 1054S	99.9			
	3M 8210	99.8			
	3M 9210	>99.9			
		# of cycles			
		1	3	5	10
Autoclave	3M 1860			99.6	99.9
	3M Aura 1870			97.4	99.6
	3M Vflex 1804S			96.5	>99.9
	AO Safety 1054S			96.9	99.4
	3M 8210			97.0	99.8
	3M 9210		99.3	99.9	93.7
		1	3	5	10
EtO	3M 1860		99.9		
	3M Aura 1870		>99.9		
	3M Vflex 1804S		99.8		
	AO Safety 1054S		99.7		
		1	2	5	10
LT-HPGP	3M 1860	92.7	88.6	60.3	
	3M Aura 1870	99.3	84.5	80.2	
	3M Vflex 1804S	96.9	92.6	88.4	
	AO Safety 1054S	91.4	90.9	84.3	
	3M 8210	92.9	66.4	57.0	
	3M 9210	98.7	94.5	89.4	
		1	3	5	10
VHP	3M 1860				99.9
	3M Aura 1870				96.7
	3M Vflex 1804S				>99.9
	AO Safety 1054S				99.9
		1	3	5	10
PAF	3M 1860				>99.9
	3M Aura 1870				>99.9
	3M Vflex 1804S				99.5
	AO Safety 1054S				95.2
		1	3	5	10
UV-C (1120 mJ/cm^2^)	3M 1860		99.6	99.4	
	3M Aura 1870		99.6	>99.9	
	3M Vflex 1804S		99.8	99.8	
	AO Safety 1054S		99.9	99.7	
	3M 8210		>99.9	>99.9	
	3M 9210		>99.9	99.9	
		1	3	5	10
Moist Heat (75°C & 22% RH X 3 hr)	3M 1860			99.8	99.9
	3M Aura 1870			>99.9	99.7
	3M Vflex 1804S			99.9	79.8
	AO Safety 1054S			99.6	99.8
	3M 8210			99.9	>99.9
	3M 9210			>99.9	99.8

Filtration testing performed at an average ambient temperature of 21.4 ± 1.4°C and 32.1 ± 6.4% relative humidity.

PAF: Peracetic acid dry fogging system, VHP = vaporous hydrogen peroxide, EtO = ethylene oxide.

LT-HPGP: Low temperature hydrogen peroxide gas plasma.

UV-C: Ultraviolet light-C radiation (254 nm wavelength).

## Discussion

The unprecedented nature of the COVID-19 pandemic has revealed previously unrecognized deficiencies in global pandemic preparedness. In particular, the depletion of single-use disposable personal protective equipment has led to prolonged use of gear far beyond standard recommendations and considerable HCW anxiety. The international shortage of N95 masks that protect against exposure to aerosolized virus, which may occur during intubation and other invasive tracheobronchial procedures, is of particular concern given the respiratory nature of the SARS-CoV-2 infections. The shortage of these masks and their use for periods beyond recommended may be part of the reason for the reported high incidence of infection seen in health care workers.

We sought to determine which decontamination techniques potentially available for use in hospitals might be suitable for the task of sterilizing a variety of N95 masks without compromising their structural or functional integrity. The perfect method would be available globally, scalable and inexpensive. In addition, the method would ideally allow for repeated decontamination cycles.

Our tests of decontamination effectiveness demonstrate that the majority of decontamination methods assessed were highly effective in sterilizing all the N95 models. No viable virus (SARS-CoV-2 where logistically possible and VSV as a surrogate if necessary) was found on any experimentally contaminated mask following autoclave, MHT, PAF, VHP, EtO gas, or LT-HPGP treatment. While most previous studies have made the assumption that such techniques would be effective at inactivating SARS-CoV-2 on N95 masks [3,9–11], this study presents SARS-CoV-2 specific data, which is crucial for evidence driven decision making.

Vesicular stomatitis virus, a bullet shaped enveloped, negative-sense RNA virus of the *Rhabdoviridae* family that commonly infects animals [12], was used as a surrogate for SARS-CoV-2 for decontamination procedures (LT-HPGP, EtO and UVCI) only available at our hospital. We could not validate SARS-CoV-2 against these three technologies because it is a Risk Group 3 virus, which cannot be manipulated outside a CL3 laboratory.

Most importantly, our results clearly show that the use of individual N95 masks can potentially be extended several-fold without degradation of functional integrity. VHP [13], PAF [14] and MHT appear to be most effective across all masks with respect to viral inactivation and retention of mask functional integrity. Recent publications have supported the possibility of using VHP and a similar hydrogen peroxide technology, Hydrogen Peroxide Vapor (HPV), for large-scale N95 decontamination strategies [9,15]. However, these studies lack inactivation data against SARS-CoV-2 or a surrogate virus. Here, we demonstrated that these methods allow at least 5 cycles of decontamination for all assessed masks without impairment of structural or functional integrity.

The potential use of VHP for N95 decontamination has been widely speculated in the context of COVID-19. Recent preprints assessing VHP to decontaminate experimentally inoculated N-95 masks have shown conflicting results in efficacy against SARS-CoV-2. In one study, complete inactivation of 4.5 logs of viable SARS-CoV-2 was demonstrated following VHP treatment^21^. In the second report, where inoculum was prepared in artificial saliva, the presence of both viral RNA and infectious virus was observed in VHP-treated mask materials [16]. The decreased efficacy of VHP decontamination in the presence of an organic soil load has been noted in a number of studies [17]. Interestingly, while a full kill was achieved using VHP in our study, which also used a soil load, a five hour cycle time was required for complete SARS-CoV-2 inactivation compared to only a single hour for VSV. This extended treatment time should be taken into consideration if turnaround times are critical in a given institution. PAF is an attractive, mobile and affordable decontamination technology [14]. Compared to VHP generating systems, with initial costs in the $75,000 CAD range and requiring annual calibration by company technicians, dry fogging systems on the other hand have significantly lower start-up costs ($5,000–10,000 CAD) and no associated annual maintenance costs. As a result, PAF may be more readily available in poorly resourced settings. This method was able decontaminate all tested masks successfully without affecting their functional integrity up to 10 cycles (maximum cycles tested). Handling and storage of extremely corrosive liquid peracetic acid and the routine cleaning requirement of the nozzles immediately after fogging to prevent clogging are the two disadvantages of this system.

Low temperature hydrogen peroxide gas plasma is commonly used in most hospitals for decontamination of high value reusable equipment such as endoscopes [18]. This study demonstrates that N95 masks do not consistently tolerate even one standard (47 min) cycle of treatment. All masks did pass fit testing after one cycle of LT-HPGP; however, half of these failed filtration testing. With 2 cycles, quantitative fit and filtration testing was impaired five of six and all 6 masks respectively; after 5 cycles, all were impaired by both testing methods. We postulate that the high concentration of liquid hydrogen peroxide (approximately 60%) and its strongly charged ionized vapor state of this device may have neutralized the filter media’s electrostatic charge, which is critical in trapping airborne particulates.

Ethylene oxide gas treatment is an older method of decontaminating materials [19]. The process is somewhat more complex than others and significant safety concerns exist in that the gas is flammable, explosive and potentially carcinogenic [20]. A prolonged period of aeration following item exposure to the gas is required to eliminate chemical residue. A very long cycle time of more than 20 hours compared to an hour or less for other decontamination methods is the result. Despite these drawbacks, some institutions in poorly-resourced settings may not have LT-HPGP or VHP. For that reason, our finding that all four mask models assessed tolerate at least 3 cycles of EtO decontamination without significant structural or functional deterioration as measured by fit and filtration testing may be useful. However, we would recommend against the use of this approach unless and until there is advanced testing to ensure that all traces of ethylene oxide and its related byproducts are entirely eliminated with sufficient aeration [21].

UV-C has been recommended as a method for decontamination/sterilization of N95 masks for potential reuse [3,22]. Virus inactivation is mediated by direct UV-C mediated damage to the viral genome. For hard surfaces, UV-C doses of <10 mJ/cm2 have been shown to be effective in generating >99% (2–3 log) reduction in viability of single strand RNA viruses [23]. The question of the required dose for sterilization of porous materials is more problematic. Suggestions of the dose required for viral inactivation efficacy have ranged from 60 mJ/cm^2^ to at least 1800 mJ/cm^2^ [3,22,24–26]. However, our results suggest that even at doses congruent with those recommended for enveloped RNA viruses, complete sterilization did not occur. Preliminary studies by others have yielded similar results with SARS-CoV-2 [27]. Based on our ancillary data using photochromatic UV-C dosimeter disks (Fig 1), we suggest the inability to totally clear viable virus stems from the fact that virus spotted in 10 μL volumes (consistent with droplets) soak into the respirator mask material deep enough protect viable virus from UV light. Further, the protein-rich nature of the soil load used in our experimental inoculum provided additional protection from UV-penetration. While there is substantial viable virus reduction with UVCI and mask integrity is well maintained, the inability to fully clear masks of viral contamination may be problematic with respect to HCW acceptance of the technique. The technique is otherwise available in most well-resourced hospitals and is scalable.

Our data show that MHT, like VHP and PAF, is highly effective for viral decontamination for all respirator models assessed and is well tolerated for repeated cycles (tested to a maximum of 10) with retention of N95 structural and functional integrity as assessed by both fit and filtration efficiency testing. The method is generally available in the community (industrial manufacturing convection ovens, bulk sterilization facilities, and industrial meat processing and livestock transport cleaning facilities) and can be relatively easily adopted in hospitals using widely available equipment (e.g. blanket warming cabinets). Another advantage is that this method is scalable and available directly within many hospital wards, allowing for local N95 mask reprocessing and easy re-use by specific individuals. A limitation is that availability is restricted to relatively well-resourced institutions. Several preliminary study publications have recently confirmed the ability of MHT of varying temperature, humidity parameters and durations to clear SARS-CoV-2 and/or preserve respirator integrity [27,28]. Similar work has been done in the past in relation to influenza virus [24,25].

The application of moist heat (pasteurization) has been used to decrease microbial pathogen counts in food products for decades. Studies clearly demonstrate that applications of >55°C heat can rapidly inactivate most viruses including human coronavirus pathogens such as SARS-CoV (SARS virus) [29] and MERS CoV (MERS virus) [30] as well as a variety of pathogenic domestic animal coronaviruses [31,32]. Available data also suggests that addition of increasing humidity enhances viral inactivation. The mechanism of virus inactivation is not entirely clear but may involve capsid and envelope disruption [33,34].

As expected, standard autoclaving using a peak temperature of 121°C to denature viral proteins results in complete elimination of viable virus. Surprisingly, however, 4 of the 6 assessed respirator mask models tolerated up to 10 cycles while maintaining structural and functional integrity according to fit testing. Although all masks maintained integrity after one autoclave cycle, the more rigid, molded 3M 1860 and 8210 models demonstrated loss of function with more than a single autoclave cycle. Interestingly, filtration remained intact in these respirators while fit testing failed suggesting the failure was due to issues of structural damage to the ability of the respirator to fit the subject. Similar findings were recently reported by Bopp *et al*, who demonstrated that the molded 1860s model failed fit testing following a single autoclave cycle of 121°C for 30 minutes while pleated masks could withstand multiple cycles [35]. Three of the 4 other layered fabric, pleated models retained integrity with up to 10 autoclave cycles (maximum number of cycles tested) with the exception of the 3M 9210 model which showed a modest decrease in filtration efficiency. These findings could be highly relevant to institutions in poorly-resourced areas of the world in that autoclaves would be expected to be available in any established hospital or major medical clinic around the world. Unfortunately, we were unable to examine the differences in mask materials and construction that might contribute to the failure of the 3M 1860 and 8210s model compared to the others due to the proprietary nature of the technology.

Single use of N95 masks for each patient encounter is ideal and recommended; unfortunately, the resource stress due to the current COVID-19 crisis has breached this ideal. According to public reporting, extended use and re-use of N95 masks has become common in hospitals in areas where SARS-CoV-2 is high. This risks functional failure of N95 masks, spread of infection to wearers and increased risk of virus transmission from health care workers to others. Our data suggests that most decontamination methods other than UVCI are effective in complete virus inactivation for at least one cycle without loss of structural integrity. However, neither LT-HPGP nor EtO gas are recommended at this time due to limited tolerance of N95 masks tested to repeat cycles, prolonged cycle times and/or potential toxicity. Our data show that PAF, VHP, MHT and autoclaving can be used to decontaminate N95 masks through at least 5 cycles without loss of function. Autoclaves can be used on a subset of N95 mask types and may be easily accessed by any healthcare institution globally when N95 mask shortages occur. MHT is also easily accessible in well-resourced settings and is scalable especially if hospital heating cabinets can be used. This simple method should also allow decontamination to remain at the ward level easing the way to re-use of masks by a single individual. Based on our data in combination with a study that showed new N95 respirator masks begin to demonstrate increasing failures after 5 cycles of fit testing (without regular use or decontamination between cycles) [7], a limit of 5 decontamination cycles using PAF, VHP, MHT or autoclave (the last for non-molded masks) decontamination seems to be an appropriate suggestion if reuse is necessary.

Although we tested the functionality of decontaminated masks via quantitative fit testing, our testing cannot take into account the respirator’s ability to withstand the rough handling that extended wear by health care workers, which stress and perspiration can inflict. Another limitation of this study is that our findings may or may not apply to other types of N95 masks. We also could not distinguish whether failure of fit or failure of filtration efficiency led to the failings of those masks upon treatment by LT-HPGP or autoclave treatments. Nonetheless, it is reassuring that the practice of appropriate decontamination and subsequent re-use of N95 mask should not pose a health risk to the already taxed health care workers.

## Conclusions

Amid the current surge of COVID19 cases, validated decontamination strategies to extend the utility of N95 masks may prove critical in the event of further global shortages. Given successful inactivation of SARS-CoV-2 combined with maintained functional integrity following 5 cycles of decontamination, peracetic acid dry fogging, VHP, autoclaving (for a subset of masks), and moist heat treatment are viable options for decontamination of most models of N95 masks.

## Supporting information

S1 Data Summary—VSV and SARS-CoV-2 inactivation results (TCID50/mL). (XLSX)
